# Have Changes in Systemic Treatment Improved Survival in Patients with Breast Cancer Metastatic to the Brain?

**DOI:** 10.1155/2008/417137

**Published:** 2008-09-23

**Authors:** Carsten Nieder, Kirsten Marienhagen, Astrid Dalhaug, Jan Norum

**Affiliations:** ^1^Radiation Oncology Unit, Medical Department - Oncology, Nordlandssykehuset HF, 8092 Bodø, Norway; ^2^Institute of Clinical Medicine, Faculty of Medicine, University of Tromsø, 9037 Tromsø, Norway; ^3^Department of Oncology, University Hospital of North Norway, 9038 Tromsø, Norway

## Abstract

Newly developed systemic treatment regimens might lead to improved survival also in the subgroup of breast cancer patients that harbour brain metastases. In order to examine this hypothesis, a matched pairs analysis was performed that involved one group of patients, which were treated after these new drugs were introduced, and one group of patients, which were treated approximately 10 years earlier. The two groups were well balanced for the known prognostic factors age, KPS, extracranial disease status, and recursive partitioning analysis class, as well as for the extent of brain treatment. The results show that the use of systemic chemotherapy has increased over time, both before and after the diagnosis of brain metastases. However, such treatment was performed nearly exclusively in those patients with brain metastases that belonged to the prognostically more favourable groups. Survival after whole-brain radiotherapy has remained unchanged in patients without further active treatment. It has improved in prognostically better patients and especially patients that received active treatment, where the 1-year survival rates have almost doubled. As these patient groups were small, confirmation of the results in other series should be attempted. Nevertheless, the present results are compatible with the hypothesis that improved systemic therapy might contribute to prolonged survival in patients with brain metastases from breast cancer.

## 1. Introduction

Whole brain radiotherapy (WBRT) continues to represent an important palliative treatment option for patients with brain metastases from breast cancer. Median overall survival typically is limited to 4–6 months [1]. However, many patients have active extracranial disease and eventually die from extracranial cancer progression. Within the last decade, several important new treatment options for metastatic breast cancer became available, for example, trastuzumab, taxane-based chemotherapy, capecitabine, and more effective aromatase inhibitors. Therefore, the possibility exists that more effective systemic treatment might lead to improved survival also in the subgroup of patients harbouring brain metastases. In order to examine this hypothesis, a matched pairs analysis was performed that involved one group of patients, which were treated after these new drugs were introduced, and one group of patients, which were treated approximately 10 years earlier.

## 2. Patients and Methods

The contemporary group included all patients with brain metastases from breast cancer, which received WBRT with 10 fractions of 3 Gy between 2000 and 2007 at the University Hospital of North-Norway. The patients (*n* = 32) were identified from the database of the Radiation Oncology facility. No patients with carcinomatous meningitis were included. Positron emission tomography for staging was not available. A historical control group treated with the same WBRT regimen between 1987 and 1997 was generated. From this larger group (*n* = 47), patients were selected for a matched pairs analysis without survival information available at the time of matching. To obtain two groups with comparable baseline characteristics, two matching criteria were used: prognostic class according to the published recursive partitioning analysis (RPA class) [3, 4] and the use of additional local treatment for relapses after WBRT, for example, radiosurgery (RS) or surgical resection. Information on primary tumour features, such as hormone receptor status and HER2 receptor status, was not available in the historical group. Information on lymphopenia [5, 6] was not available at all. We used the Kaplan-Meier method to generate actuarial survival curves. These were compared with the log rank test. Wilcoxon- and Kruskal-Wallis tests were used to compare the baseline characteristics between the two groups. A *P*-value <.05 was considered statistically significant.

## 3. Results

Table 1 shows the patient characteristics for the contemporary group and the matched group of 32 historical patients. As can be seen, the groups were balanced for the established prognostic factors. More patients in the contemporary group had received chemotherapy before diagnosis of brain metastases. Time to development of brain metastases was significantly longer. The fact that more patients in the historical group were recorded to have had single brain metastases apparently was not related to increased use of magnetic resonance imaging in recent years.

The majority of patients in the contemporary group were lymph node positive, hormone receptor negative, and HER-2 positive. Neither hormone receptor status nor HER-2 status was statistically significant prognostic factor in this group (*P* > .3). Only 4 patients had not received systemic treatment, 2 had received endocrine treatment only, and 26 had received chemotherapy before development of brain metastases (5 patients had 3 different lines of chemotherapy). Twelve patients remained without systemic treatment after WBRT, 5 received endocrine treatment only, 11 one line of chemotherapy, and 4 at least 2 lines of chemotherapy. Three patients received trastuzumab-containing treatment after WBRT. As this was a matching criterion, 3 patients in each group received RS or surgical resection for progressive brain metastases. As a striking difference, only 8 patients in the historical group received additional chemotherapy after WBRT (endocrine treatment unfortunately was not recorded).

In the contemporary group, 60% of the patients achieved at least a partial remission of their brain metastases based on imaging after WBRT (reduction in largest diameter of each lesion by at least 50% without development of new lesions). At 6 months after WBRT, 59% of the patients were progression-free in the central nervous system. These data are not available for the historical group. Median survival from first diagnosis of breast cancer was 102 months in contemporary patients with initial T1/2 N0 disease and 47.5 months in patients with more advanced disease (*P* < .05). These figures are higher than in historical patients (60.9 versus 34.7 months). Table 2 demonstrates that the recent survival improvement from the start of WBRT is entirely derived from RPA class II patients, while no obvious progress was achieved in RPA class III. Analysis of class I was not meaningful, as this group included only 2 patients. Only 2 of the contemporary patients in RPA class III received chemotherapy after WBRT (versus one patient in the historical group). Patients without active treatment after WBRT had a median survival of 3.5 versus 3.2 months, that is, no improvement over time. For patients with active treatment, median survival also remained stable (9.0 versus 7.9 months), while the 1-year survival rate improved from 25 to 43%. The small group (*n* = 6) of patients that received the most aggressive treatment, defined as trastuzumab-containing regimens and/or brain salvage treatment, had a median survival of 15.4 months (4 patients survived >1 year).

## 4. Discussion

This matched pairs analysis was performed with two groups of patients, which received identical local treatment for their brain metastases and were well balanced for the known prognostic factors age, KPS, extracranial disease status, graded prognostic assessment (GPA) group, and RPA class. Unlike the newly developed GPA, RPA class has been confirmed as prognostic factor in patients with primary breast cancer in several studies (Table 3). In order to obtain enough historical patients for the matching procedure, the treatment period was extended back to the year 1987. The recently suggested prognostic factor lymphopenia [5, 6] has not been routinely assessed in our department and could, therefore, not be included. A further point that needs to be mentioned is the lack of information on hormone receptor status, HER-2 status, and endocrine treatment in the historical patient group, which was treated before 1998. Thus, imbalances regarding these parameters, which might have influenced the survival results to some degree, cannot be ruled out. As demonstrated in Table 4, the prognostic impact of hormone receptor and HER-2 status is difficult to interpret at this time, as the published studies reported inconsistent results and are all limited in size. None of the patients in the historical group received trastuzumab, taxanes, capecitabine, or new-generation aromatase inhibitors.

The first interesting finding was that the interval from initial breast cancer treatment to brain metastases has increased by 18 months, that is, 53%. Median interval was longer than previously reported in the literature (Table 3). Accordingly, increased overall survival from first diagnosis of breast cancer was evident, in particular in patients with initial T1/2 N0 disease. Such results might be explained by a change in the biology of the disease. However, the numbers of patients with node-positive disease, receptor-negative disease, and HER-2 positive disease between 2001 and 2007 argues against this explanation. Especially HER-2 overexpression and hormone receptor negativity appear to influence the risk of brain metastases development [16]. In fact, the data favour changes in treatment regimens as a more likely explanation. The use of systemic chemotherapy has increased over time, both before and after the diagnosis of brain metastases. However, such treatment was performed nearly exclusively in those patients with brain metastases that belonged to the prognostically more favourable groups (better RPA class). Whether changes in systemic treatment are the only explanation for the marked survival improvement in RPA class II in the present study is difficult to assess. Other factors might potentially influence the results. It is, for example, possible that more patients that previously would have been assigned to RPA class I would now be assigned to class II, based on better methods for detection of extracranial metastatic disease. In addition, RPA class II is a quite inhomogeneous group of patients with large potential differences, for example, in extent of CNS involvement and KPS.

In the present analysis, survival after WBRT has remained unchanged in patients without further active treatment. It has improved in prognostically better patients and especially patients that received active treatment, where the 1-year survival rates have almost doubled. As these patient groups were small, confirmation of the results in other series should be attempted. When looking at the literature, the series that is best comparable to our own contemporary patients is the one reported by Le Scodan et al. [6], which provides almost identical survival data (Table 3). The series that included the oldest data (back to 1984) [7], is very close to our own historical group. Bartsch et al. confirmed that patients without systemic treatment have significantly poorer median survival (5 months versus 10 months in patients with systemic treatment) [8]. These authors also found that intensified local therapy of brain metastases improved the outcome. Lee et al. confirmed the value of both intensified local and systemic treatment in a series of 198 patients [17]. Other nonrandomized series suggest that RS might lead to better local control and survival than that reported from WBRT series [18]. The impact of chemotherapy on brain control is less clear [19], although recent data suggest that capecitabine might be able to induce remission of central nervous system metastases from breast cancer [20]. Taken together, the present results and the literature overview are compatible with the hypothesis that improved systemic therapy might contribute to longer survival in patients with brain metastases from breast cancer, which qualify for active systemic therapy in addition to upfront WBRT.

## Figures and Tables

**Table 1:** Patient characteristics.

	Contemporary group, *n* = 32	Historical group, *n* = 32	*P*-value
Median age, range	55 yrs., 34–81	53 yrs., 29–72	>.1
Median KPS, range	70%, 50–90	70%, 30–90	>.1
Median time interval*	52 mo., 3–216	34 mo., 7–118	<.05
% single brain metastasis	16	34	<.05
% with MRI scan of the brain	44	41	>.1
% uncontrolled primary	3	0	>.1
% without extracranial metastases	9	9	>.1
% RPA class I versus II versus III	6 : 59 : 34	6 : 56 : 38	>.1
% GPA group I versus II versus III versus IV	0 : 0 : 62 : 38	0 : 6 : 56 : 38	>.1
Median GPA score	1.5	1.5	>.1
% chemotherapy before brain metastases diagnosis	81	69	<.1
% T1/2 N0 versus T3/4 N0 versus N+	25 : 6 : 69	19 : 6 : 75	>.1
% with HR positive tumour	42	not available	
% with HER-2 positive tumour	69	not available	

KPS: Karnofsky performance status, * from breast cancer diagnosis to brain metastases, MRI: magnetic resonance imaging, RPA: recursive partitioning analysis, GPA: graded prognostic assessment (Sperduto et al. [2]: 0-1 point (most unfavourable) defined as group IV, 1.5–2.5 points defined as group III, 3 points defined as group II, 3.5–4 points defined as group I), HR: hormone receptor.

**Table 2:** Survival results for the two groups.

	Contemporary group, *n* = 32	Historical group, *n* = 32	*P*-value (log-rank test)
Median survival	5.0 mo.	3.6 mo.	
1-year survival	31%	19%	<.1
Median survival RPA class II	9.0 mo.	3.4 mo.	
1-year survival RPA class II	42%	22%	<.05
Median survival RPA class III	2.4 mo.	3.1 mo.	
1-year survival RPA class III	18%	8%	>.1

**Table 3:** Comparison with the literature, median WBRT dose 30 Gy in all studies.

	Own contemporary patients	Mahmoud-Ahmed et al. [7]	Claude et al. [5]	Bartsch et al. [8]	Le Scodan et al. [6]
Treated	2000–2007	1984–2000	1991–2001	1994–2004	1998–2003
*n*	32	116	120	174	117
Upfront surgery/RS	no	no	yes	yes	no
Median age	55 y.	50 y.	54 y.	55 y.	53 y.
Median interval	52 m.	22.5 m.	38 m.	35 m.	39 m.
Extracranial met.	91%	68%	80%	unknown	94%
Previous Ctx	81%	unknown	84%	unknown	79%
HR positive	42%	unknown	66%	unknown	48%
Median survival	5.0 m.	4.2 m.	5.0 m.	7.0 m.	5.0 m.
1-year survival	31%	17%	25%	30%	28%
Median survival class II	9.0 m.	6.1 m.	9.0 m.	unknown	8.0 m.
Median survival class III	2.4 m.	1.7 m.	3.0 m.	unknown	3.0 m.

RS: radiosurgery, Ctx: chemotherapy, HR: hormone receptor.

**Table 4:** Prognostic impact of hormone receptor and HER-2 status.

	*n*	Prognostic impact of hormone receptor status	Prognostic impact of HER-2 status
Claude et al. [5]	120	none	not examined
Bartsch et al. [8]	174	none	none
Le Scodan et al. [6]	117	receptor negative significantly worse	none
Nam et al. [9]	126	receptor negative significantly worse	HER-2 negative significantly worse
Kirsch et al. [10]	95	not examined	HER-2 negative significantly worse*
Eichler et al. [11]	83	none	HER-2 negative significantly worse^⋀^
Melisko et al. [12]	112	receptor negative significantly worse	none
Harputluoglu et al. [13]	144	none	none
Park et al. [14]	125	none	HER-2 positive significantly worse
Church et al. [15]	86	not examined	HER-2 negative significantly worse*
Own contemporary group	32	none	none

^⋀^ 80% of HER-2 overexpressing cases received trastuzumab after diagnosis of brain metastases. * The difference in survival was limited to patients with HER-2 overexpressing cancer treated with trastuzumab after diagnosis of brain metastases.
